# Associations of Muscle Strength with Central Aspects of Pain: Data from the Knee Pain and Related Health in the Community (KPIC) Cohort

**DOI:** 10.3390/jpm13101450

**Published:** 2023-09-29

**Authors:** Daniel F. McWilliams, Bin Yue, Stephanie L. Smith, Joanne Stocks, Michael Doherty, Ana M. Valdes, Weiya Zhang, Aliya Sarmanova, Gwen S. Fernandes, Kehinde Akin-Akinyosoye, Michelle Hall, David A. Walsh

**Affiliations:** 1 Pain Centre Versus Arthritis, University of Nottingham, Nottingham NG7 2RD, UKstephanie.smith2@nottingham.ac.uk (S.L.S.); joanne.stocks@nottingham.ac.uk (J.S.); michael.doherty@nottingham.ac.uk (M.D.); ana.valdes@nottingham.ac.uk (A.M.V.); weiya.zhang@nottingham.ac.uk (W.Z.); aliyasarmanova@gmail.com (A.S.); michelle.hall@nottingham.ac.uk (M.H.); david.walsh@nottingham.ac.uk (D.A.W.); 2 NIHR Nottingham Biomedical Research Centre, Nottingham University Hospitals NHS Trust, Nottingham NG7 2UH, UK; 3 Academic Rheumatology, Division of Injury, Recovery and Inflammation Sciences, School of Medicine, University of Nottingham, Nottingham NG5 1PB, UK; 4 Eighth Affiliated Hospital, Sun Yat-sen University, Shenzhen 518033, China; 5 Centre for Sports, Exercise, and Osteoarthritis Research Versus Arthritis, University of Nottingham, Nottingham NG7 2UH, UK; 6 Bristol Medical School, University of Bristol, Bristol BS8 1TH, UK; 7 Division of Physiotherapy Education, School of Medicine, University of Nottingham, Nottingham NG7 2UH, UK; 8 Sherwood Forest Hospitals NHS Foundation Trust, Mansfield NG17 4JL, UK

**Keywords:** musculoskeletal pain, quantitative sensory testing, osteoarthritis, muscle weakness, pain

## Abstract

Knee pain is associated with lower muscle strength, and both contribute to disability. Peripheral and central neurological mechanisms contribute to OA pain. Understanding the relative contributions of pain mechanisms to muscle strength might help future treatments. The Knee Pain and related health In the Community (KPIC) cohort provided baseline and year 1 data from people with early knee pain (n = 219) for longitudinal analyses. A cross-sectional analysis was performed with baseline data from people with established knee pain (n = 103) and comparative data from people without knee pain (n = 98). Quadriceps and handgrip strength indicated local and general muscle weakness, respectively. The indices of peripheral nociceptive drive were knee radiographic and ultrasound scores. The indices associated with central pain mechanisms were Pressure Pain detection Threshold (PPT) distal to the knee, and a validated self-report Central Aspects of Pain Factor (CAPF). The associations were explored using correlation and multivariable regression. Weaker quadriceps strength was associated with both high CAPF and low PPT at baseline. Year 1 quadriceps weakness was predicted by higher baseline CAPF (β = −0.28 (95% CI: −0.55, −0.01), *p* = 0.040). Weaker baseline and year 1 handgrip strength was also associated with higher baseline CAPF. Weaker baseline quadriceps strength was associated with radiographic scores in bivariate but not adjusted analyses. Quadriceps strength was not significantly associated with total ultrasound scores. Central pain mechanisms might contribute to muscle weakness, both locally and remote from the knee.

## 1. Introduction

Chronic knee pain is a major cause of disability, mainly in association with knee osteoarthritis (OA) [1]. Pain and weakness are often concordant [2,3,4], suggesting shared mechanisms. Mechanistic explanations of OA pain highlight both peripheral (joint pathology) and central mechanisms [1,5]. Understanding contributions from peripheral and central mechanisms could lead to new targeted treatments for pain and disability.

Although OA can be asymptomatic, chronic pain is its primary symptom [1]. Important pain mechanisms are peripheral nociception and neuronal sensitisation, facilitated by knee structural changes or inflammation, and central mechanisms (e.g., central sensitisation) [5,6]. OA is associated with radiographic evidence of bony spurs (osteophytes) and focal loss of cartilage (joint space narrowing, JSN), and, sometimes, synovial hypertrophy, effusion, and increased Doppler blood flow evident using ultrasonography. Pain is associated with knee radiographic changes and synovitis [7], and with the indices of central sensitisation (quantitative sensory testing (QST) and self-report) [8]. A discordance exists between indices of peripheral or central mechanisms and pain severity. Knee pain is commonly experienced without radiographic changes, and individuals with radiographic OA may be pain-free [9]. Furthermore, 10% to 34% of people experience persistent knee pain after total joint replacement surgery for knee OA, suggesting that the removal of nociceptive drive might not always eliminate pain [10]. These suggest that, as well as nociceptive drive, central mechanisms may contribute to knee pain [5,6].

People with knee pain commonly report weakness and the giving way of their knees, despite having no detectable instability [11]. Knee pain may reduce quadriceps’ voluntary contractions [12]. Previous studies which have investigated pain severity, rather than pain mechanisms, reported lower muscle strength in symptomatic knees without radiographic changes, compared with asymptomatic knees with severe radiographic changes [2,3,4]. The inhibition of muscle function may persist after the resolution of acute pain, suggesting that mechanisms can become independent of ongoing pain [13]. Indeed, exercise might not be able to modify some measurements of pain sensitivity, such as onset analgesia [14].

Pain sensitivity can be examined using QST. In people with knee pain, low Pressure Pain detection Thresholds (PPT), at sites distal to or remote from the affected joint, might indicate changed central nervous system pain processing [1,15]. Akin-Akinyosoye et al. identified a unitary factor associated with central aspects of pain (termed the Central Aspects of Pain Factor (CAPF) in this study). CAPF is based on a self-report questionnaire comprising items that were associated with multiple aspects of this central dysfunction. CAPF was associated with PPT distal to the painful knee [8,16]. CAPF showed a Cronbach’s alpha of 0.80, maps onto a unitary trait and fits a single factor model, and behaves as an index of central pain augmentation in individuals with knee pain [8,16,17]. In the Nottingham Knee Pain and related health In the Community (KPIC) study, CAPF showed good measurement properties, validity against pain severity, and predictive utility for future knee pain [8,17].

We hypothesised that the association between knee pain and low muscle strength primarily may be due to central rather than peripheral pain mechanisms, which might lead to systemic weakness. Central mechanisms could drive widespread reductions in muscle strength and might predict lower future strength at multiple sites including those remote from the knee joint. Here, we report associations of muscle strength with central and peripheral pain mechanism indices in KPIC.

## 2. Materials and Methods

### 2.1. Study Population

**Figure 1:**
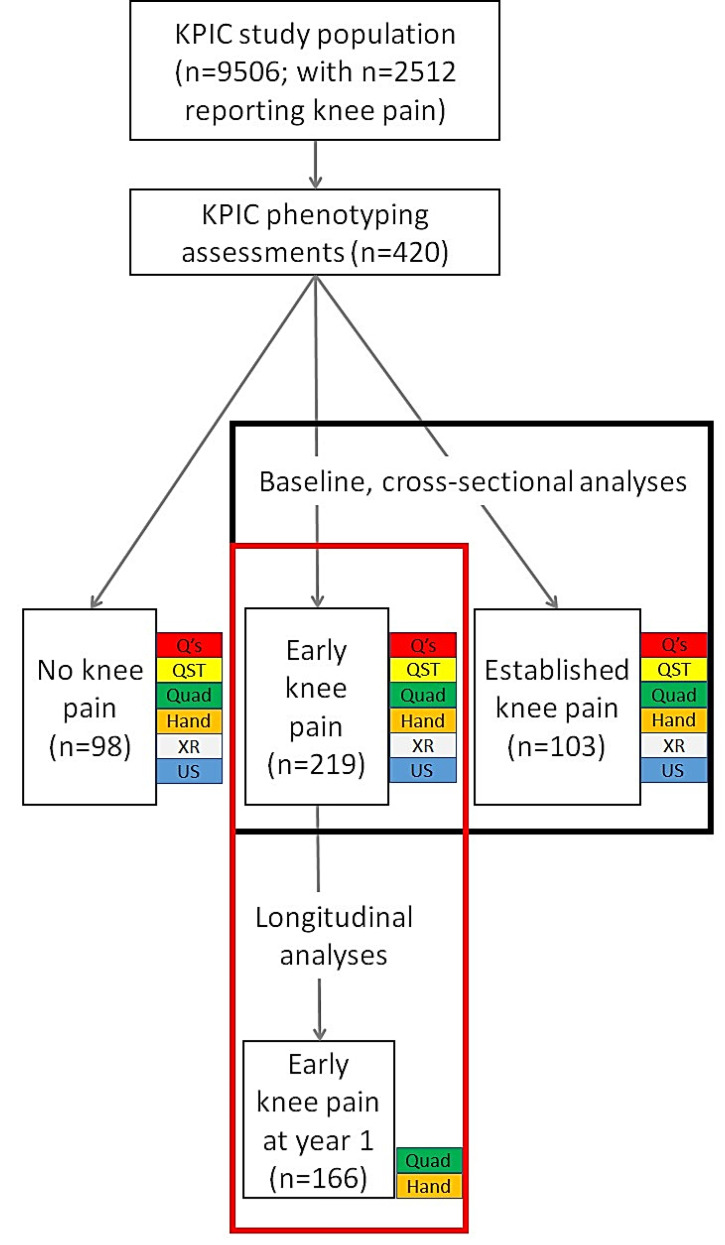
Flow diagram showing the numbers of participants at each stage of this study and which groups were used for different analyses of the associations with muscle strength. Numbers of participants at each stage and in each group are shown. Data collections modalities used for the reported analyses in this study are shown at each stage (baseline in black box and longitudinal analysis in red box). Q’s = questionnaires, demographics and clinical information; QST = pressure pain detection threshold; Quad = quadriceps strength; Hand = handgrip strength; XR = radiography; US = ultrasound scan.

The data analysed were from the KPIC prospective cohort study [18], which was designed to investigate mechanisms and associations of knee pain in community-dwelling adults, and included the measurement of muscle strength. Data from KPIC have been included in previous publications [7,8,17,18,19,20,21]. KPIC recruitment is detailed in Figure 1, and 420 participants took part in the clinical and imaging assessments used for this study. Participants for KPIC were invited from adults in the East Midlands region of the UK, with inclusion criteria being age ≥40 years who were registered with general practices, regardless of knee pain status. The exclusion criteria included those unable to give informed consent and people with terminal illness. Pregnant women were also excluded from clinical assessments. Participants were selected for clinical assessments based on current knee pain status, irrespective of radiographic findings. “Early knee pain” was defined as pain reported to have commenced within the past 3 years regardless of pain severity. “Established knee pain” was moderate to severe knee pain that had lasted more than 3 years [7]. “No knee pain” participants were selected to be age- and sex-matched to early knee pain cases [18]. At baseline, 420 participants provided data, of whom 219 had early knee pain, 103 had established knee pain, and 98 had no knee pain. Follow-up data at 1 year were only provided by participants within the early knee pain group (n = 166; Figure 1). The study was approved by the Nottingham Research Ethics Committee 1 (NREC Ref: 14/EM/0015) and registered (clinicaltrials.gov portal: NCT02098070).

### 2.2. Self-Report Knee Pain Severity

A community-based questionnaire survey was designed to capture detailed information about the participants [18]. Average 4-week knee pain severity was assessed using a 0–10 numerical rating scale (NRS): “In the past month, on average, how intense was the pain in your most painful knee rated on a 0–10 scale, where 0 is ‘no pain’ and 10 is ‘pain as bad as could be’? (That is, your usual pain at times you were experiencing pain)”.

The index knee referred to the only (unilateral) or most painful (bilateral) knee, selected based on response to the item “Which knee do you/did you experience the pain in?” If both, the most painful knee was selected based on the item “which overall is the worst knee?” A knee was selected using a random number sequence if both knees were equally painful.

### 2.3. Central Aspects of Pain Factor

The CAPF was derived as previously published [8,17] by summating scores from 8 items from questionnaires selected using expert consensus for relevance to central pain mechanisms and statistical association with PPT distal to the most painful knee. CAPF items addressed, or were derived from questionnaires that addressed, neuropathic-like pain, catastrophizing, depression, anxiety, sleep disturbance, fatigue, cognitive impact, and widespread pain distribution [8,16,17,22,23,24,25,26,27,28,29]. Earlier validation using factor analysis informed the re-scaling of item scores to achieve a range of 0–3 for each item, providing a summated CAPF score from 0 to 24 [17]. Summated scores were also generated for validated scales from which CAPF items were derived; specifically, HADS anxiety, HADS depression, painDETECT, and Pain Catastrophizing Scale (PCS) [30,31,32].

### 2.4. Pressure Pain Detection Thresholds

The protocol for PPT was modified from that used in a previously published study [33]. PPT was measured using a hand-held pressure algometer, comprising a probe with a circular end (1 cm^2^) (Somedic AB, Norra Mellby, Sweden) that was connected to a computer [18]. The probe was applied perpendicular to the proximal tibia (5 cm distal to the tibial tuberosity of each leg) and applied pressure was gradually increased at a standardized rate of 30 kPa/s. Participants were asked to press a button when the feeling of pressure changed to pain, following which the pressure was immediately released. Familiarisation procedures on an index finger were performed prior to PPT data collection. The whole PPT testing process was repeated 3 times, with 2 min recovery between each algometer application, and the average was used in analyses. PPT data were collected by 3 observers, and their reliability has been published previously (inter-rater reliability (concordance correlation coefficient, CCC) = 0.51, intra-rater reliability, CCC = 0.60 [17]).

### 2.5. Radiographic Assessment

Bilateral tibiofemoral and patellofemoral radiographs were obtained using a standardized protocol involving a standing semiflexed postero-anterior view (with a Rosen template) and supine 30° flexion skyline views [18]. The Nottingham logically devised line drawing atlas (NLDA) was used to grade osteophyte (0 to 3) and JSN (−1 to +3) for each joint compartment (medial tibiofemoral, lateral tibiofemoral, and patellofemoral compartments) [18]. JSN was modified by treating −1 as a 0 (i.e., “joint space widening”, which was usually due to narrowing elsewhere within the joint, was scored the same as “no joint space narrowing”) [34]. A total radiographic severity score for the index knee was calculated by summating scores for the medial and lateral tibiofemoral and medial and lateral patellofemoral JSN and osteophytes (possible score range = 0 to 36). Osteoarthritis classification was reported using the method of Kellgren and Lawrence [35] for the tibiofemoral and patellofemoral joint compartments separately. Radiographs were scored by a single observer (GSF), and with good reliability (kappa values ≥ 0.78 for intra-rater and inter-rater agreements [7]).

### 2.6. Ultrasound Imaging

Both knee joints were imaged using ultrasound using a Toshiba Aplio SSA-770A (Toshiba, Minato, Tokyo, Japan) machine with a multi-frequency (7–12 MHz) linear array transducer. The measurement was performed with knee flexion of about 20–30°, including suprapatellar recess, medial, and lateral tibiofemoral spaces. The longitudinal axis was used to measure the maximal synovial thickness and effusion depth in millimetres [36]. The Power Doppler assessment focused on areas of synovial hypertrophy and a Positive Power Doppler signal that provided information on vascularity was recorded as being neither absent or present [18]. The continuous ultrasound measurements were summated to produce a total ultrasound measure. Ultrasound data were collected by 2 observers and their reliability has been published previously (inter-observer agreement between 2 observers was κ = 0.44 for effusion and κ = 0.61 for synovial hypertrophy. The intra-observer agreement for effusion was κ = 0.50 [7]).

### 2.7. Quadriceps Strength

The isometric maximum voluntary contraction (MVC) was assessed by using a ‘Nicholas Manual Muscle Tester’ (Lafayette Instruments, Lafayette, IN, USA) handheld dynamometer. Participants sat upright on a stable, unarmed surface, with thighs horizontal, knees at 90°, and feet off the ground. The dynamometer was placed at the anterior distal tibia 5cm above the malleoli (no strapping was used). Participants were asked to “push the muscle tester with as much force as possible in an attempt to extend the lower leg”, with resistance provided by the researcher. The average value of 3 repeats for each leg was recorded [18,37].

### 2.8. Handgrip Strength

A JAMAR hydraulic hand dynamometer (Lafayette Instruments, Lafayette, IN, USA) was used to assess dominant handgrip strength. Participants were asked to sit upright in a stable, unarmed, four-legged chair with thighs horizontal and knees at 90°. The dominant hand was selected. Participants kept their flexed elbow tight to the waist, upper arm vertical, lower arm horizontal, and the other arm relaxed. The device was squeezed as hard as possible on setting #2 [38] and then released. The average value of 3 repeats was recorded [18]. Muscle strength data were collected by 3 observers, and their reliabilities have been published previously (CCCs ranging from 0.64 for inter-rater to 0.94 for intra-rater reliability [7]).

### 2.9. Statistical Analysis

To compare differences between study groups, Chi-squared, Fishers exact, one-way ANOVA or independent samples *t*-tests were performed as appropriate. Descriptive statistics for continuous variables were reported as mean and standard deviation (SD), categorical variables as frequencies, and variables with skewed distribution as the median and interquartile range (IQR). Spearman’s rho correlation coefficient was applied with 95% CI calculated through 1000 bootstrap draws. Comparisons between correlation coefficients were performed using R within the RStudio software (RStudio 2022.07.1+554, rstudio.com, access date 24/7/2022) and the *cocor* package [39,40]. Changes in muscle strength from baseline to 1 year were analysed using Wilcoxon signed rank test. Data from people with no knee pain were compared at baseline to other study groups to show differences from normal strength, the direction of change at 1 year, and its association with knee pain. The no knee pain group was not included in the analyses which investigated knee pain severity, as there was no pain reported in these people. At baseline, correlations and regressions with muscle strength were for all participants with knee pain (early knee pain and established knee pain groups), whereas only participants in the early knee pain group provided data for longitudinal analyses. CAPF and PPT were used as indices associated with central pain mechanisms (Figure 1). Variables were z-transformed prior to regression analysis for the calculation of standardized coefficient β, presented with 95% Confidence Interval (95% CI). Natural log transformation was used to reduce skewness in the data prior to regression analysis. Multivariable regression analysis was performed with muscle strength (baseline or 1 year) as the dependent variable (quadriceps was the primary analysis in the current study and handgrip strength was secondary), with independent variables of CAPF, PPT, NLDA radiographic scores, ultrasound scores, study group (for cross-sectional analysis at baseline, where more than 1 group was included), age, sex (female = 2, male = 1), BMI, and baseline muscle strength (for longitudinal analysis at 1 year only). In order to reduce the number of statistical comparisons, both sexes were examined within the same statistical models with adjustments for sex included. No adjustments were made for baseline pain in analyses that assessed the potential effects of CAPF on muscle strength, because increased pain severity is one of the candidate pathways for effect. *p* < 0.05 was taken to indicate statistical significance. Analyses used complete case data, using IBM SPSS Statistics software version 27 (IBM, Chicago, IL, USA). Power calculations were performed using G*power software version 3.1 (Kiel University, Germany).

## 3. Results

### 3.1. Participant Characteristics

The demographic and clinical characteristics are summarised in Table 1. At baseline, 420 participants provided data, of whom 219 had early knee pain. Longitudinal data were provided at one-year follow-up for muscle strength by 74% (164/219) of the participants with early knee pain. At year 1, median (IQR) quadriceps strength was decreased to 14 (12 to 18) kg (*p* < 0.001 when compared to baseline), and handgrip strength was not significantly different to 24 (17 to 32) kg (*p* = 0.35 compared to baseline).

### 3.2. Associations of Pain Mechanism Indices with Quadriceps Strength

The bivariable associations at baseline in people with knee pain (early and established groups combined) are presented in Supplementary Table S1 and Figure S1. At baseline, pain severity was associated with both CAPF (rho = 0.56, 95% CI 0.47, 0.64) and PPT (rho = −0.22, 95% CI −0.32, −0.09), and also with radiographic severity (NLDA, rho = −0.15, 95% CI −0.29 to −0.07) and total ultrasound synovitis score (rho = 0.13 95% CI 0.02 to 0.23 Associations of weaker quadriceps strength with higher CAPF or lower PPT (greater sensitivity) were of similar strength to associations with pain (|rho| values 0.32 to 0.40). At baseline, stronger correlations with quadriceps strength were found for CAPF (rho = −0.40, 95% CI −0.49 to −0.28) than for either radiographic severity (rho = −0.15, 95% CI −0.26, −0.04) or ultrasound synovitis score (rho = −0.06, 95% CI −0.11 to 0.12) (z = 2.9, *p* = 0.003 and z = 3.8, *p* < 0.001, respectively). Higher scores for each CAPF item were also associated with weaker quadriceps strength (Supplementary Table S2). In the multivariable regression model, baseline quadriceps strength in people with knee pain remained significantly associated with both baseline CAPF and PPT, but not with radiographic or ultrasound indices of peripheral nociceptive drive (Table 2).

The bivariable associations between baseline variables and year 1 muscle strength are shown in Supplementary Table S2. A weaker year 1 quadriceps strength was associated with higher baseline CAPF (rho = −0.26, 95% CI 0.07, 0.44). The associations with higher baseline pain (rho = −0.10, 95% CI −0.25, 0.05), lower PPT (greater sensitivity, rho = 0.15, 95% CI −0.01, 0.30), higher radiographic score (NLDA, rho = −0.11, 95% CI −0.23, 0.07) or total ultrasound score (rho = 0.04, 95% CI −0.15, 0.15) were not statistically significant. The baseline and year 1 quadriceps strength measurements were associated with each other (rho = 0.40, 95% CI 0.28, 0.52). The correlation with quadriceps strength was significantly stronger for CAPF than for ultrasound (z = 3.3, *p* = 0.001). The multivariable regression model of year 1 quadriceps strength in people with early knee pain at baseline is summarised in Table 3. A weaker year 1 quadriceps strength remained significantly associated with higher baseline CAPF after adjustment for age, sex, BMI, and the other indices associated with peripheral nociceptive drive and central pain mechanisms. A weaker year 1 quadriceps strength was also associated with higher baseline CAPF, but not other indices, in regression analyses that replaced total ultrasound score with effusion, hypertrophy or Doppler scores, or replaced NLDA with JSN or osteophyte scores (Supplementary Tables S4–S8).

### 3.3. Associations of Knee Pain and Pain Mechanisms with Handgrip Strength

Associations of dominant handgrip strength were investigated as an index of systemic muscle strength. The bivariable associations at baseline in people with knee pain (early and established groups combined) are presented in Supplementary Table S1. At baseline, weaker handgrip strength was associated with weaker quadriceps strength (rho = 0.57, 95% CI 0.48, 0.64), and with more severe knee pain (rho = −0.16, 95% CI −0.26, −0.05), higher CAPF score (−0.28, 95% CI −0.39, −0.17), and lower PPT (greater sensitivity, rho = 0.35, 95% CI 0.24, 0.47). In the multivariable regression model of baseline data from people with knee pain, weaker handgrip strength remained significantly associated with higher CAPF, lower PPT (greater sensitivity), older age, and female sex (Table 2).

The bivariable associations between baseline variables and year 1 handgrip strength are shown in Supplementary Table S2. A weaker year 1 handgrip strength was associated with higher baseline CAPF (rho = −0.34, 95% CI −0.48, −0.19) and lower PPT (greater sensitivity, rho = 0.19, 95% CI 0.02, 0.34). Year 1 handgrip strength was not significantly associated with baseline knee pain severity. Higher scores for individual CAPF items were also associated with weaker handgrip strength (Supplementary Table S3). The baseline and year 1 handgrip strength measurements were associated with each other (rho = 0.86, 95% CI 0.81 to 0.90). In the multivariable regression model, a weaker year 1 handgrip strength remained significantly associated with a weaker baseline handgrip strength, higher baseline CAPF, older age, and female sex, after adjustment for BMI and PPT (Table 3).

## 4. Discussion

We found that high CAPF and lower PPT distal to the painful knee were associated with quadriceps weakness both cross-sectionally and longitudinally, in both bivariable and (for CAPF) multivariable models. The associations of quadriceps strength with CAPF or PPT were of similar magnitude to its associations with pain severity, and greater than with radiography- or ultrasound-detected knee joint pathology. Furthermore, the indices of central pain were associated with widespread muscle weakness, as evidenced by reduced handgrip strength. Baseline CAPF was associated longitudinally with a worse prognosis for muscle strength, both local to and distant from the index joint, and more strongly than baseline pain. CAPF might reflect the central mechanisms that could influence muscle strength over and above their effects on pain.

Our study extends previous reports that quadriceps weakness is associated with concurrent knee pain [2,3,4], to indicate that this association might be more due to central mechanisms than to OA pathology local to the knee. Furthermore, these baseline indices were more strongly predictive of year 1 weakness than baseline pain severity itself. If central mechanisms prove to be a cause of weakness, interventions which address them might help people with knee pain to maintain or increase their strength.

Previous studies of muscle strength in knee OA have focused mainly on associations with radiographic OA change or diagnosis [2,3,12,41,42,43], or synovitis [44,45,46,47]. Each reflects joint pathology that we confirm is associated with pain, consistent with indices of peripheral pain mechanisms. Each might have additional effects on quadriceps strength through biomechanical mechanisms [46,47]. However, quadriceps weakness was more strongly associated with pain severity than with radiographic OA severity [2,3,4], suggesting that radiographic change alone does not explain quadriceps weakness in OA. We confirmed the cross-sectional associations of weaker quadriceps strength with radiographic OA severity and ultrasound evidence of synovitis. However, these associations became non-significant when adjusting for age, sex, and central pain mechanisms, indicating possible confounding by non-structural factors to the best of our knowledge. Furthermore, baseline radiographic or ultrasound scores did not significantly predict progressive muscle weakness.

CAPF and PPT were also associated with current knee pain [8,15,16,17], supporting their inclusion as indices associated with central pain mechanisms. We found that these indices were more strongly associated with muscle weakness than were radiographic and ultrasound indices of peripheral pathology. Furthermore, higher baseline CAPF in people with early knee pain was associated with muscle weakness at one year. Predictions of weakness by CAPF were found even after adjusting for baseline muscle strength, suggesting that it might contribute to progressive muscle weakness. Our findings suggest that central mechanisms could be more important causes of weakness in people with knee pain than are direct effects of structural or inflammatory change in the joint.

The central mechanisms may include reflex interactions between pain and motor pathways, leading to the inhibition of muscle activation or voluntary contraction [48]. PPT distal to the painful knee has been taken as an index of central sensitisation [15]. CAPF was associated with muscle strength, even after adjusting for PPT, suggesting that its contribution to muscle strength depends on mechanisms additional to those measured by PPT. The CAPF, measured using self-report items [8,16], might indicate psychological or neurophysiological mechanisms that could influence muscle strength. The psychological correlates of CAPF, such as depression and catastrophizing, might predispose to lower levels of physical activity with consequent sarcopenia, impaired muscle quality, and reductions in muscle strength [48]. Our data lead us to suggest that targeting central pain mechanisms might help increase muscle strength.

We found that knee pain and central pain mechanisms were also associated with handgrip weakness. In a previous study, people with unilateral knee OA had weakened contralateral thigh muscles, indicated by lower isometric extensor and flexor strength on the pain-free side, compared with bilateral pain-free controls [49]. Widespread weakness suggests systemic effects on muscle strength, rather than purely local effects at the affected knee, or regional effects such as altered gait [50]. Widespread weakness contributes to frailty, a vulnerability state that is increasingly prevalent in our ageing population [51]. Our study raises possible shared contributions from central mechanisms to both pain and frailty. The mechanisms of such association are unknown but could include the well-innervated fascia, which is a source of current research interest [52]. However, fascial sensitivity might be best measured by the direct stimulation from PPT [53], rather than by CAPF, so this is unlikely to be regulating this association exclusively. However, other mechanisms derived from the fascia have not been ruled out.

Our study has several limitations. Our longitudinal design enabled us to capture dynamic changes, thereby increasing our potential to detect important associations during the development of established knee pain. However, this entailed using data from only a subgroup of the baseline study population, introducing possible selection bias and limiting statistical power. The longitudinal analysis appears to have used data from a representative sample of the study group. We cannot rule out weak (rho < 0.2) but important effects of PPT, ultrasound or radiography scores. Our measurement of CAPF was via a self-report questionnaire and might be influenced by reporting patterns or by measurement properties. Each item within the CAPF has previously been shown to be associated with central pain mechanisms, and the CAPF forms a unitary trait. Associations with muscle weakness were found across all 8 items, although we cannot completely exclude the possibility that the observed association between CAPF and muscle weakness might be driven by one or more of these separate characteristics, rather than any shared dysfunction of the central nervous system. Furthermore, we cannot completely exclude the indirect effects of CAPF on muscle weakness, for example, mediated through lower activity levels. We have not excluded possible bidirectional effects between pain and muscle weakness. The indices of pain mechanisms used in this study have limited sensitivity, and other measures such as contrast-enhanced MRI [44] might have revealed important peripheral mechanisms contributing to muscle weakness. Our models explained 27% of the variance in quadriceps muscle strength, and 41% of the variance in handgrip strength (Table 2), leaving a large proportion of the variance unexplained. Unmeasured factors might affect muscle strength, for example, activity levels, genetic constitution, comorbidities, and pharmacological and non-pharmacological management approaches used by KPIC participants [7]. Comorbidities, in particular, might have an important effect, as they are known to be associated with worse bodily pain and also could influence muscle weakness. However, the construct “comorbidities” encompasses a large and complex range of conditions, and might warrant separate study. Populations with larger changes in muscle strength might reveal additional predictors of prognosis. Changes in muscle strength as small as 5–10% might be clinically meaningful [54], and weak associations might yet represent important biological mechanisms. Quadriceps strength measured with the Nicholas Manual Muscle Tester may be subject to researcher bias or drift over the course of the study, as the assessor holds the instrument and resists the force applied by the participant. Measures of handgrip strength might also have been affected by instrumental settings and a lack of external support during measurements, although setting #2 for the JAMAR dynamometer has been shown to have good reliability in other studies [38]. Indeed, the coordination and control required during our handgrip testing might also be related to neuromuscular control, which we hypothesise might be related to CAPF. Our measures of pain severity and central pain mechanisms depended on self-report by participants, which may be affected by recall bias, reporting style, and other confounders. The CAPF was developed in the KPIC cohort [17], with some validation in other datasets [16]. Our current findings are consistent with CAPF representing a distinct construct, but further research is required to fully understand its mechanistic underpinning. Controlled interventional studies, for example, targeting central pain mechanisms, are required to demonstrate causality.

## 5. Conclusions

In conclusion, we found that indices of central pain mechanisms were associated with and predicted widespread muscle weakness in people with knee pain, whereas the associations between indices of peripheral nociceptive mechanisms and muscle strength might be explained by factors such as total pain severity (from all causes), disability or others. Interventions to increase muscle strength have traditionally focused on exercises and physical activity, although people with chronic knee pain may find these difficult to engage with, and long-term adherence is often suboptimal. Our data lead us to suggest that future research could assess knee pain, and, particularly, central pain mechanisms, and determine whether pharmacological or non-pharmacological therapies might further improve muscle strength and reduce disability.

## Figures and Tables

**Table 1:** Baseline demographics and clinical characteristics of participants.

	Early Knee Pain Group		Established Knee Pain (n = 103)	No Knee Pain (n = 98)
	Early Knee Pain (n = 219)	Participants with Muscle Strength Data at 1 Year (n = 164)		
**Demographics and lifestyle**				
Age (years), mean (SD)	60 (9)	61 (9)	60 (10)	61 (10)
Female % (n)	61% (134)	62% (102)	61% (63)	60% (59)
BMI (kg/m^2^), mean (SD)	28.8 (5.6)	28.5 (5.2)	31.5 (6.5)	26.9 (4.3)
**Muscle strength**				
Quadriceps strength (kg), median (IQR)	16.2 (11.1–23.1)	16.8 (11.7–23.9)	11.4 (6.8–15.6)	16.5 (12.0–18.6)
Handgrip strength (kg), median (IQR)	24.0 (17.3–32.2)	24.0 (17.5–32.6)	22.3 (15.0–30.3)	25.2 (18.6–34.7)
**Pain**				
Average 4-week knee pain, 0–10, mean (SD)	5.3 (2.5)	5.1 (2.5)	8.0 (1.7)	Not measured
**Central mechanisms**				
PPT (proximal tibial; kPa), median (IQR)	399 (278–540)	411 (298–576)	316 (217–488)	377 (270–581)
CAPF, mean (SD)	6.9 (4.1)	6.2 (3.8)	10.7 (4.3)	Not measured
**Peripheral mechanisms**				
Joint space narrowing, median (IQR)	0 (0–2)	1 (0–2)	2 (0–4)	0 (0–1)
Osteophyte, median (IQR)	2 (0–6)	2 (0–6)	7 (2–15)	1 (0–2)
NLDA X-ray (range 0–36), median (IQR)	2 (0–8)	2 (0–8)	8 (2–14)	1 (0–3)
K&L Score ≥2 Tibiofemoral Joint % (n)	23% (48)	23% (38)	46% (46)	5% (5)
Ultrasound Doppler signal present % (n)	3% (7)	4% (7)	3% (3)	0% (0)
Ultrasound effusion (mm) median (IQR)	3.5 (1.7–5.9)	3.5 (1.1–5.7)	5.6 (3.2–8.4)	2.7 (1.9–4.9)
Ultrasound synovial hypertrophy (mm) median (IQR)	0 (0–3.4)	0 (0–3.4)	3.7 (0–6.6)	0 (0–0)
Total ultrasound, median (IQR)	3.9 (2.0–9.4)	3.9 (2.0–8.9)	9.1 (3.9–14.8)	2.8 (1.9–6.10)

Baseline characteristics of the 3 groups within KPIC. CAPF: central aspects of pain factor score; PPT: pressure pain threshold; NLDA: Nottingham logically devised line drawing atlas for radiographic severity of OA; K&L: Kellgren and Lawrence score for OA.

**Table 2:** Multivariable regression showing cross-sectional associations at baseline of quadriceps or hand grip strength with demographic variables and indices of pain mechanisms.

Dependent Variable	Independent Variable	β	95% CI	*p*
**Quadriceps strength**	**CAPF**	**−0.24**	**−0.37 to −0.11**	**<0.001**
	**PPT**	**0.14**	**0.01 to 0.26**	**0.031**
	NLDA	−0.09	−0.22 to 0.05	0.195
	Ultrasound total	−0.04	−0.17 to 0.10	0.595
	Age	−0.05	−0.18 to 0.08	0.442
	**Sex**	**−0.28**	**−0.40 to −0.16**	**<0.001**
	BMI	0.06	−0.07 to 0.18	0.351
	Study group	−0.14	−0.28 to 0.00	0.057
**Handgrip strength**	**CAPF**	**−0.21**	**−0.33 to −0.08**	**0.001**
	**PPT**	**0.19**	**0.08 to 0.30**	**<0.001**
	**Age**	**−0.15**	**−0.26 to −0.05**	**0.005**
	**Sex**	**−0.47**	**−0.57 to −0.37**	**<0.001**
	BMI	0.08	−0.03 to 0.18	0.145
	Study group	−0.03	−0.15 to 0.09	0.620

Multivariable linear regression for muscle strength at baseline. The quadriceps strength model, n = 207, explained 27% of variance in quadriceps muscle strength. The handgrip strength model, n = 211, explained 41% of variance in handgrip strength. Statistical significance is highlighted in bold. CAPF: central aspects of pain factor score; PPT: pressure pain detection threshold; NLDA: Nottingham logically devised line drawing atlas; study groups were coded ordinally as 1 = early knee pain, 2 = established knee pain. Statistical significance is indicated with bold.

**Table 3:** Multivariable regression of year 1 quadriceps or hand grip strength with baseline indices of pain mechanisms and other variables.

Dependent Variable	Baseline Independent Variable	β	95% CI	*p*
**Year 1 quadriceps strength**	**CAPF**	**−0.28**	**−0.55 to −0.01**	**0.040**
	PPT	0.07	−0.14 to 0.28	0.515
	NLDA	0.01	−0.27 to 0.29	0.934
	Ultrasound total	0.16	−0.12 to 0.44	0.257
	Age	−0.23	−0.45 to 0.03	0.055
	Sex	−0.17	−0.41 to 0.07	0.173
	BMI	0.04	−0.22 to 0.29	0.785
	Quadriceps strength	0.22	−0.02 to 0.46	0.071
**Year 1 handgrip strength**	**CAPF**	**−0.19**	**−0.33 to −0.05**	**0.008**
	PPT	0.03	−0.08 to 0.14	0.589
	**Age**	**−0.19**	**−0.32 to −0.06**	**0.005**
	**Sex**	**−0.25**	**−0.40 to −0.10**	**0.001**
	BMI	−0.04	−0.17 to 0.09	0.554
	**Handgrip strength**	**0.69**	**0.49 to 0.89**	**<0.001**

Linear regression models for muscle strength at 1 year in the early knee pain group. Dependent variables year 1 quadriceps strength and year 1 handgrip strength. Models explained 57% of variance for quadriceps strength (n = 111) and 63% of handgrip strength (n = 118). Statistical significance is highlighted in bold. CAPF: central aspects of pain factor score; PPT: pressure pain detection threshold; NLDA: Nottingham logically devised line drawing atlas. Retrospective power calculations showed a >95% power for the model to detect an association different from the null for both dependent variables. Statistical significance is indicated with bold.

## Data Availability

Data access requests should be directed to W.Z. in the first instance. The KPIC study was registered on the clinicaltrials.gov portal: NCT02098070. The study analysis plan for this manuscript was not preregistered.

## Supplementary Materials

The following supporting information can be downloaded at: https://www.mdpi.com/article/10.3390/jpm13101450/s1, Figure S1: Scatterplots showing associations with quadriceps strength at baseline. Table S1: Correlations between baseline variables; Table S2: Correlation of baseline variables with year 1 muscle strength; Table S3: Examining the component items of the Central aspects of pain factor and their association with muscle strength; Table S4: Regression for year 1 quadriceps strength with hypertrophy replacing total ultrasound score; Table S5: Regression for year 1 quadriceps strength with effusion replacing total ultrasound score; Table S6: Regression for year 1 quadriceps strength with doppler replacing total ultrasound score; Table S7: Regression for year 1 quadriceps strength with joint space narrowing (JSN) replacing total radiography score; Table S8: Regression for year 1 quadriceps strength with osteophyte (OST) replacing total radiography score.
