# Supplementary material for: Associations of Muscle Strength with Central Aspects of Pain: Data from the Knee Pain and Related Health in the Community (KPIC) Cohort
Source: J Pers Med. 2023 Sep 29;13(10):1450. doi: 10.3390/jpm13101450 (PMC10608698; doi:10.3390/jpm13101450)
Supplement: Supplementary file 1 [file jpm-13-01450-s001.zip › jpm-2588014-supplementary.pdf]

Supplementary Information

Supplement table S1. Correlations between baseline variables

		Quad strength	Handgrip strength	PPT	CAPF	Radiography (NLDA)	Osteophyte	JSN	Ultrasound	US effusion	US hypertrophy	US doppler	Average 4 week knee pain	BMI	Sex
Handgrip strength	rho	**.570****													
	p	**<0.001**													
	N	**311**													
PPT	rho	**.324****	**.353****												
	p	**<0.001**	**<0.001**												
	N	**271**	**273**												
CAPF	rho	**-.397****	**-.284****	**-.204****											
	p	**<0.001**	**<0.001**	**0.002**											
	N	**258**	**262**	**225**											
Radiography (NLDA)	rho	**-.150****	**-.118***	**-.125***	0.075										
	p	**0.009**	**0.038**	**0.041**	0.231										
	N	**307**	**309**	**270**	256										
Osteophyte	rho	-0.108	-0.06	**-.132***	0.071	**.961****									
	p	0.058	0.292	**0.03**	0.259	**<0.001**									
	N	307	309	**270**	256	**310**									
JSN	rho	**-.150****	**-.193****	-0.087	0.06	**.815****	**.661****								
	p	**0.009**	**0.001**	0.153	0.338	**<0.001**	**<0.001**								
	N	**307**	**309**	270	256	**310**	**310**								
Ultrasound	rho	-0.059	0.014	-0.105	0.012	**.513****	**.504****	**.418****							
	p	0.304	0.804	0.084	0.845	**<0.001**	**<0.001**	**<0.001**							
	N	309	311	271	258	**307**	**307**	**307**							
US effusion	rho	-0.062	0.012	-0.077	-0.007	**.529****	**.505****	**.447****	**.951****						
	p	0.276	0.838	0.208	0.914	**<0.001**	**<0.001**	**<0.001**	**<0.001**						
	N	309	311	271	258	**307**	**307**	**307**	**312**						
US hypertrophy	rho	-0.024	0.009	**-.144***	0.033	**.450****	**.457****	**.349****	**.882****	**.718****					
	p	0.669	0.88	**0.018**	0.6	**<0.001**	**<0.001**	**<0.001**	**<0.001**	**<0.001**					
	N	309	311	**271**	258	**307**	**307**	**307**	**312**	**312**					

US Doppler	rho	**.121***	0.11	**.125***	**-.128***	0.035	0.024	0.024	**.207****	**.179****	**.186****				
	p	**0.032**	0.052	**0.039**	**0.04**	0.545	0.675	0.675	**<0.001**	**0.001**	**0.001**				
	N	**312**	314	**274**	**260**	310	310	310	**312**	**312**	**312**				
Average 4 week knee pain	r	**-.359****	**-.158****	**-.215****	**.562****	**.246****	**.243****	**.199****	**.128***	**.131***	0.106	**-.120***			
	p	**<0.001**	**0.005**	**<0.001**	**<0.001**	**<0.001**	**<0.001**	**<0.001**	**0.024**	**0.02**	0.061	**0.033**			
	N	**312**	**317**	**274**	**264**	**310**	**310**	**310**	**312**	**312**	312	**315**			
BMI	rho	**-.123***	-0.077	**-.185****	**.290****	**.136***	**.165****	0.059	0.099	0.089	0.104	**-.203****	**.226****		
	p	**0.035**	0.178	**0.003**	**<0.001**	**0.02**	**0.004**	0.315	0.09	0.126	0.074	**<0.001**	**<0.001**		
	N	**296**	304	**260**	**251**	**295**	**295**	295	296	296	296	**299**	**303**		
Sex	rho	**-.405****	**-.690****	**-.270****	**.155***	-0.038	-0.1	0.089	**-.169****	**-.151****	**-.161****	**-.151****	0.109	**.117***	
	p	**<0.001**	**<0.001**	**<0.001**	**0.012**	0.507	0.078	0.118	**0.003**	**0.008**	**0.004**	**0.007**	0.052	**0.042**	
	N	**312**	**320**	**274**	**264**	310	310	310	**312**	**312**	**312**	**315**	319	**306**	
Age	rho	0.057	**-.123***	0.01	**-.140***	**.341****	**.292****	**.358****	**.172****	**.174****	**.153****	0.101	**-.152****	**-.173****	**-.158****
	p	0.317	**0.028**	0.871	**0.023**	**<0.001**	**<0.001**	**<0.001**	**0.002**	**0.002**	**0.007**	0.073	**0.006**	**0.002**	**0.004**
	N	312	**320**	274	**264**	**310**	**310**	**310**	**312**	**312**	**312**	315	**319**	**306**	**322**

Spearmans rho, p and n= for baseline variables. Significance is shown in bold. **Correlation is significantly at the 0.01 level(2-tailed). *Correlation is significant at the 0.05 level(2-tailed). *Post hoc* analysis indicated ≥82% power to detect a correlation coefficient ≥0.18 at baseline in bivariate analyses. BMI: body mass index; CAPF: central aspects of pain factor; PPT: pressure pain threshold (proximal tibial); JSN=joint space narrowing; US=ultrasound; sex: male=1 and female=2.

Supplementary Figure S1:

Scatterplots showing associations with quadriceps strength at baseline

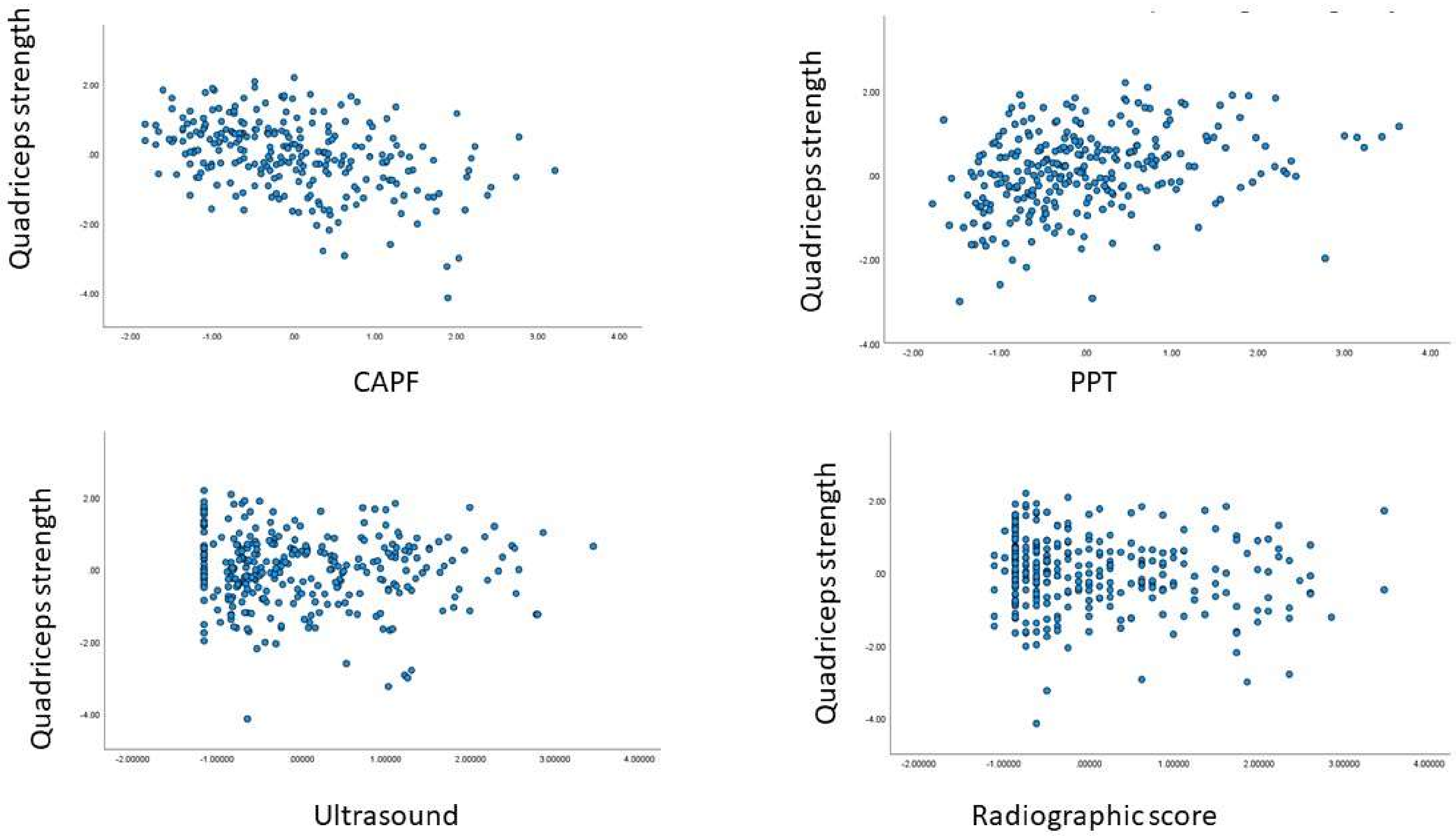

Supplement table S2: Correlation of baseline variables with year 1 muscle strength

		Quad strength 1 year	Handgrip strength 1 year
Quad strength baseline	rho	**.323****	
	p	**<0.001**	
	N	**158**	
Handgrip strength 1 year	rho	**.486****	
	p	**<0.001**	
	N	**162**	
Handgrip strength baseline	rho	**.515****	**.864****
	p	**<0.001**	**<0.001**
	N	**161**	**165**
PPT	rho	0.149	**.189***
	p	0.066	**0.019**
	N	153	**153**
CAPF	rho	**-.263****	**-.340****
	p	**0.003**	**<0.001**
	N	**126**	**128**
Radiography	rho	-0.114	-0.051
	p	0.153	0.524
	N	158	158
Osteophyte	rho	-0.088	0.003
	p	0.269	0.972
	N	158	158
JSN	rho	-0.093	-0.057
	p	0.244	0.474
	N	158	158
Ultrasound	rho	0.044	0.133
	p	0.581	0.095
	N	159	159
US effusion	rho	0.015	0.095
	p	0.847	0.235
	N	159	159
US hypertrophy	rho	0.064	0.154
	p	0.424	0.053
	N	159	159
US Doppler	rho	-0.059	0.136
	p	0.46	0.086
	N	161	161
Average 4 week knee pain	r	-0.099	-0.093
	p	0.212	0.236
	N	162	164

BMI	rho	-0.083	-0.141
	p	0.304	0.076
	N	155	159
Sex	rho	**-.448****	**-.682****
	p	**<0.001**	**<0.001**
	N	**162**	**166**
Age	rho	-0.139	**-.159***
	p	0.077	**0.041**
	N	162	**166**

**Correlation is significant at the 0.01 level(2-tailed). *Correlation is significant at the 0.05 level(2-tailed). Post hoc analysis indicated ≥80% power to detect r≥0.26 between baseline variables and year 1 quadriceps strength in bivariate analyses. BMI: body mass index; CAPF: central aspects of pain factor; PPT: pressure pain threshold (proximal tibial); JSN=joint space narrowing; US=ultrasound; sex: male=1 and female=2.

Supplement table S3: Examining the component items of the Central aspects of pain factor and their association with muscle strength.

Each individual item from the CAPF was assessed for its Spearmans correlations with muscle strength at baseline and at 1 year follow up. 95% CI were calculated from 1000 bootstrap draws.

		Baseline		1 year	
		**Quad**	**Handgrip**	**Quad**	**Handgrip**
**Anxiety**	rho	-0.20	-0.19	-0.11	-0.14
	95% CI	(-0.31 to -0.09)	(-0.30 to -0.07)	(-0.26 to 0.05)	(-0.29 to 0.01)
	p	<0.001	0.001	0.173	0.068
**Depression**	rho	-0.19	-0.05	-0.07	-0.10
	95% CI	(-0.29 to -0.08)	(-0.16 to 0.06)	(-0.21 to 0.08)	(-0.25 to 0.06)
	p	0.001	0.400	0.349	0.225
**Neuropathic-like**	rho	-0.28	-0.22	-0.25	-0.29
	95% CI	(-0.39 to -0.17)	(-0.32 to -0.10)	(-0.40 to -0.11)	(-0.42 to -0.14)
	p	<0.001	<0.001	0.002	<0.001
**Cognition**	rho	-0.33	-0.19	-0.14	-0.14
	95% CI	(-0.42 to -0.23)	(-0.30 to -0.08)	(-0.29 to 0.01)	(-0.30 to 0.02)
	p	<0.001	0.001	0.074	0.069
**Widespread pain**	rho	-0.12	-0.10	-0.18	-0.17
	95% CI	(-0.23 to -0.01)	(-0.21 to 0.01)	(-0.32 to -0.02)	(-0.33 to -0.01)
	p	0.030	0.068	0.025	0.034
**Catastrophizing**	rho	-0.24	-0.07	-0.09	-0.07
	95% CI	(-0.35 to -0.13)	(-0.18 to 0.04)	(-0.24 to 0.07)	(-0.21 to 0.09)
	p	<0.001	0.210	0.253	0.402
**Sleep**	rho	-0.34	-0.18	-0.22	-0.22
	95% CI	(-0.45 to -0.24)	(-0.30 to -0.06)	(-0.40 to -0.04)	(-0.36 to -0.06)
	p	<0.001	0.002	0.010	0.009
**Fatigue**	rho	-0.29	-0.19	-0.10	-0.19
	95% CI	(-0.39 to -0.19)	(-0.29 to -0.08)	(-0.25 to 0.05)	(-0.34 to -0.03)
	p	<0.001	0.001	0.202	0.013

Supplementary table S4: Regression for year 1 quadriceps strength with hypertrophy replacing total ultrasound score

	B	95% Confidence Interval for B		p
		Lower Bound	Upper Bound	
Age	-0.23	-0.47	0.00	0.053
Female	-0.18	-0.41	0.06	0.146
BML	0.04	-0.21	0.29	0.753
Baseline quad strength	0.21	-0.03	0.44	0.084
CAPF	-0.29	-0.56	-0.03	0.032
PPT	0.07	-0.14	0.29	0.501
US hypertrophy	0.17	-0.15	0.48	0.295
NLDA	0.04	-0.23	0.30	0.789

Supplementary table S5: Regression for year 1 quadriceps strength with effusion replacing total ultrasound score

		95% Confidence Interval for B		
	B	Lower Bound	Upper Bound	p
Age	-0.22	-0.46	0.01	0.059
Female	-0.18	-0.41	0.06	0.142
BMI	0.03	-0.22	0.28	0.825
Baseline quad strength	0.22	-0.02	0.46	0.069
CAPF	-0.28	-0.55	-0.01	0.040
PPT	0.06	-0.15	0.27	0.568
US effusion	0.13	-0.11	0.37	0.287
NLDA	0.02	-0.26	0.30	0.900

Supplementary table S6: Regression for year 1 quadriceps strength with doppler replacing total ultrasound score

	B	95% Confidence Interval for B		p
		Lower Bound	Upper Bound	
Age	-0.22	-0.45	0.01	0.059
Female	-0.24	-0.47	-0.02	0.034
BMI	0.01	-0.24	0.26	0.949
Baseline quad strength	0.20	-0.04	0.43	0.095
CAPF	-0.32	-0.58	-0.05	0.019
PPT	0.04	-0.16	0.24	0.706
US doppler	-0.08	-0.25	0.10	0.393
NLDA	0.11	-0.12	0.35	0.353

Supplementary table S7: **Regression for year 1 quadriceps strength with joint space narrowing (JSN) replacing total radiography score**

	B	95% Confidence Interval for B		p
		Lower Bound	Upper Bound	
Age	-0.25	-0.48	-0.01	0.040
Female	-0.18	-0.42	0.06	0.146
BMI	0.03	-0.22	0.27	0.831
Baseline quad strength	0.22	-0.02	0.46	0.067
CAPF	-0.30	-0.57	-0.03	0.032
PPT	0.06	-0.15	0.27	0.572
US	0.13	-0.13	0.39	0.326
JSN	0.10	-0.18	0.39	0.477

Supplementary table S8: Regression for year 1 quadriceps strength with osteophyte (OST) replacing total radiography score

	B	95% Confidence Interval for B		p
		Lower Bound	Upper Bound	
Age	-0.23	-0.46	0.01	0.055
Female	-0.16	-0.40	0.08	0.176
BMI	0.04	-0.21	0.29	0.76
Baseline quad strength	0.22	-0.02	0.46	0.070
CAPF	-0.28	-0.55	-0.01	0.041
PPT	0.07	-0.14	0.28	0.508
US	0.17	-0.11	0.44	0.225
OST	-0.01	-0.29	0.27	0.962
